# Association between textual and pictorial warnings on tumbac (waterpipe tobacco) boxes and motivation to quit waterpipe smoking among Lebanese and Iraqi adolescents

**DOI:** 10.1186/s12887-024-04649-7

**Published:** 2024-03-08

**Authors:** Diana Malaeb, Bassam Abdul Rasool Hassan, Ali Haider Mohammed, Sinan Subhi Farhan, Omar Abdulwahid Al-Ani, Abir Sarray El Dine, Feten Fekih-Romdhane, Sahar Obeid, Souheil Hallit

**Affiliations:** 1 College of Pharmacy, Gulf Medical University, Ajman, United Arab Emirates; 2 Department of Pharmacy, Al Rafidain University College, Baghdad, Iraq; 3 School of Pharmacy, Monash University Malaysia, Bandar Sunway, Malaysia, Selangor 47500 Malaysia; 4 Department of Anesthesia, College of Medical Science Technology, University of Mashreq, Baghdad, Iraq; 5 School of Pharmacy, Lebanese International University, Beirut, Lebanon; 6 The Tunisian Center of Early Intervention in Psychosis, Department of psychiatry “Ibn Omrane”, Razi hospital, Manouba, 2010 Tunisia; 7 Faculty of Medicine of Tunis, Tunis El Manar University, Tunis, Tunisia; 8 Social and Education Sciences Department, School of Arts and Sciences, Lebanese American University, Jbeil, Lebanon; 9 School of Medicine and Medical Sciences, Holy Spirit University of Kaslik, P.O. Box 446, Jounieh, Lebanon; 10 Applied Science Research Center, Applied Science Private University, Amman, Jordan

**Keywords:** Motivation to quit, Pictorial warning, Textual warning, Waterpipe, Adolescents

## Abstract

**Background:**

Waterpipe tobacco smoking has increased tremendously at a global level among all age groups, particularly young people. Previous studies have examined the impact of waterpipe tobacco pictorial health warnings on adults but scarce studies were done on adolescents. The aim of this study was to assess the association of textual versus pictorial warnings on tumbac boxes and the motivation to quit waterpipe smoking among adolescents located in two Eastern Mediterranean countries Lebanon and Iraq.

**Methods:**

A cross-sectional study was conducted between May and November 2022, involving 294 adolescents waterpipe smokers from Lebanon and Iraq. The questionnaire included the Lebanese Waterpipe Dependence Smoking-11, the Depression, Anxiety and Stress Scale, the Waterpipe Harm Perception Scale, Waterpipe Knowledge Scale, Waterpipe Attitude Scale, the Fagerstrom Test for Nicotine Dependence, and the Motivation to Stop Scale.

**Results:**

When adjusting the results over confounding variables, the results showed that compared to finding the warnings to stop smoking not efficacious at all, adolescents who find the warnings moderately (aOR = 2.83) and very (aOR = 6.64) efficacious had higher motivation to quit. Compared to finding the warnings not increasing their curiosity for information about how to stop waterpipe smoking at all, participants who confessed that warnings increased their curiosity a little (aOR = 2.59), moderately (aOR = 3.34) and very (aOR = 3.58) had higher motivation to quit. Compared to not considering changing the tumbac brand if the company uses pictorial warnings, adolescents who would consider changing the tumbac brand (aOR = 2.15) had higher motivation to quit.

**Conclusion:**

Pictorial and textual warnings on waterpipe packs were associated with higher motivation to stop waterpipe smoking. Public health education programs for this purpose seem warranted.

## Introduction

Waterpipe tobacco smoking (WTS) has tremendously increased at a global level among all age groups, particularly among young people [1]. For many centuries, WTS has been extensively practiced in the Eastern Mediterranean Region with a further increase in recent years [2]. According to a recent review, the rate of WTS is highest among adults in the Eastern Mediterranean region, and among youth the prevalence was around 10% in the European region [3]. The prevalence of waterpipe or tobacco consumption in the Eastern Mediterranean ranged between 22.5 and 36.9% among students [4].

The wide use of WTS in the market can be attributed to the introduction of different flavors and the social norms and beliefs which favor its consumption [5]. Other positive attributes are the belief that the practice of waterpipe smoking induces fun, increases pleasure, and promotes relaxation [6]. In addition, most waterpipe smokers falsely believe that waterpipe is a safer option than cigarettes mediated by filtering water when the smoke bubbles pass through the tube, lower temperature of the waterpipe smoke compared to cigarette smoke, and the availability of aromatic smells and pleasant taste [5]. Also, the incorrect public perception of reduced harm induced by waterpipe is possibly driven by the advancement of global communication particularly the internet, and the lack of enforcement of waterpipe tobacco control policies [7, 8]. In addition, the wide spread practice of WTS among youth is explained by the limited awareness and knowledge about the detrimental health risk associated with smoking in general and WTS in particular [9, 10]. At young age, waterpipe smokers were able to identify the non-communicable diseases associated with WTS as cardiovascular, respiratory, and cancer-related harmful consequences [8]. However, the majority of waterpipe smokers were not able to identify the negative consequences related to mental and cognitive effects as well as its impact on productivity, pregnancy, and neonates [11]. This discrepancy in the knowledge about WTS can be interpreted by the limited availability of comprehensive information about its harmful consequences [11]. Numerous data support the fact that social anxiety, stress and depression were found to be predictors of waterpipe smoking among adolescents [12, 13]. According to previous studies conducted in the Arab world, waterpipe smoking is highly linked to mental disorders since smokers are more susceptible to develop depressive symptoms compared to nonsmokers [12, 14].

One of the most effective population-based strategies to enhance the practice of smoking cessation is through communicating and spreading the awareness about the harms of smoking [15]. Health Warning Labels (HWLs) are considered an efficient mean to communicate and spread the potential health risks associated with smoking. According to the Framework Convention on Tobacco Control (FCTC), implementing the HWLs on tobacco packages might reduce morbidity and mortality associated with tobacco [16]. HWLs exert numerous advantages that help achieve smoking cessation through raising health knowledge about the risk perceptions, triggering the motivation to quit, and preventing smoking initiation among non-smokers [17]. A recent study conducted in the United States showed that waterpipe smokers non-exposed to HWLs had a higher total puff volume, more puffs, shorter interpuff intervals a higher smoking puffs consumption, more satisfaction, enhanced puff/taste liking, and lower perception to the harmful effects of WTS as compared to those exposed to HWLs [18]. Therefore, HWLs raised the attention and the cognitive reactions associated with smoking risks and considered an aid strategic tool in smoking cessation [17, 19–24]. According to FCTC, HWLs should cover at least 30–50% of tobacco packaging, with pictorial HWLs being superior to text-only [16]. In addition to the HWLs and pictorial warning, enhancing the knowledge about the health risks, improving perception, and shifting into a more favorable attitude towards quitting smoking increase the motivation to quit [25]. Thus, raising knowledge about the detrimental health consequences serves as a powerful motivator to quit smoking and protect health [26]. Improving health information about the risks associated with smoking is considered an avenue to better informed decisions about quitting smoking and maximizing health behaviors [27]. There is supporting evidence that misperception about the harms of smoking tobacco products is responsible for a low motivation to quit smoking and impede efforts to cessation behaviors [28]. Furthermore, improving perception and attitude involves recognizing the importance of both short- and long-term benefits of quitting smoking and can positively impact knowledge about the linked health risks [28]. It has been documented that smokers who actively seek health information to improve their personal attitudes and beliefs enhance their abilities to make informed decisions and engage in smoking cessation behaviors [29]. Overall, studies support that motivation to quit smoking is related to high levels of knowledge about the health harms of tobacco, positive attitude towards smoking cessation, and favorable perception of the benefits associated with stopping smoking.

The widespread prevalence of waterpipe smoking among adolescents in the Eastern Mediterranean region raises a great concern and alarming sign since it is associated with devastating consequences [2, 30, 31]. The most efficacious interventional plan designed for smoking cessation involves different assessments, including the evaluation of perception towards smoking and motivation to quit [32]. In light of the increased evidence pointing towards the consequences of waterpipe smoking, there is a need to assess the level of knowledge and evaluate its associated factors among waterpipe adolescents [11].

Currently, Lebanon does not mandate pictorial warnings; it requires the addition of only textual health warnings on tobacco products packaging [33]. According to the Lebanese law Number 174 “Tobacco Control and Regulation of Tobacco Products’ Manufacturing, Packaging and Advertising”, it is prohibited to provide tobacco products including waterpipe unless its cover encompasses a notice about the risks and effects of the use on health [34]. In Lebanon, albeit the release of the law, and despite the effectiveness of pictorial warning was proven to increase motivation to quit smoking, it is still not systematically implemented on waterpipe products [35]. As for Iraq, although Iraq laws required the implementation of both pictorial and textual warnings to cover the front and back surfaces of all tobacco product packaging, the details of warnings required on packaging for products other than cigarettes are applied [36]. Although Iraq had issued the laws of implementation since 2019, there are no studies that assessed the impact of the pictorial warnings on smoking cessation or the motivation to quit smoking [37]. Various studies that were conducted among smokers in universities both in the US and Arab countries concluded that HWLs increase motivation to quit [38–42]. While one study assessed the impact of textual warning on tobacco boxes on the motivation and intention to quit on Lebanese adults [42] and another one among Jordanian adults and minors [43], yet there is no study that has examined this association among adolescents in Lebanon. Thus, our study targeted the adolescent population, since it is a critical phase where people tend to engage in risky behaviors, exhibit high substance abuse, and exhibit the highest prevalence of waterpipe smoking [3, 44, 45]. Furthermore, there are different factors that trigger the smoking practice among adolescents, which include peers pressure that encourage cigarette and waterpipe smoking and parental smoking behavior that foster the smoking environment [45].

Our study was conducted in Lebanon and Iraq since according to the Global Youth Tobacco Survey, the prevalence of tobacco smoking was not the same where 15.7% of the students in Iraq [46] and 36.2% in Lebanon currently smoke tobacco products [47]. Furthermore, Iraq had recently witnessed an increase in the smoking prevalence to 20% and the prevalence of tobacco products is 11% among youth [48]. Thus, we studied the association of both pictorial and textual warnings on tobacco products on the motivation to quit smoking among adolescents from two different Arab countries with vast differences in smoking rate and different cultural backgrounds. Therefore, this study aimed to assess the association of textual versus pictorial warnings on tumbac boxes and the motivation to quit waterpipe smoking among adolescents located in two Eastern Mediterranean countries Lebanon and Iraq.

## Methods

### Study design and participants

Cross-sectional research involving 294 teen waterpipe smokers was carried out between May and November 2022. Participants were gathered from a variety of carefully chosen restaurants located throughout Lebanon and Iraq. Those locations were picked at random from those in regions where cafés and restaurants are common. Every day at peak times (after 7 PM), research assistants went there to make sure the most waterpipe smokers were present. All waterpipe smokers were initially approached in cafes, and asked if they would be willing to take part in the research and complete the survey. If they agreed to participate, the link was sent to them via social applications. All data collection was web-based, and no data collection was done face-to-face in cafes. Participants had to be under 18 years old and current waterpipe smokers (defined as presently smoking ≥ 1 waterpipe per week). Participants who declined to participate in the study were excluded.

### Minimal sample size calculation

Using the G-power software v.3.0.10 (option: multiple regression, R^2^ deviation from zero), a minimum sample of 205 was deemed necessary, based on a R^2^ deviation of 10% (chosen arbitrarily), an alpha error of 5%, a power of 80% and a maximum of 20 variables to be entered in the final model.

### Questionnaire

The digital questionnaire was distributed to all participants via a Google form link. The study objectives were explained to each participant before being handed the anonymous, self-administered questionnaire. On average, the questionnaire required approximately 15 min to be completed by participants. The pre-tested questionnaire was adapted to the local Arabic language (the native language of Lebanon and Iraq). The study information and objectives were provided in the introductory paragraph, while insisting on participants to take their parents’ approval to participate. Before beginning the survey, all respondents reported having had their parents’ consent to take part in the study.

The first part of the questionnaire tackled sociodemographic characteristics (age, gender), the Household Crowding Index (HCI) [49] reflecting the socioeconomic status of the family was also included. It is the ratio of the number of people living in the house over the number of rooms (excluding the kitchen and the bathrooms) and the Physical Activity Index was calculated by multiplying the intensity by the frequency by the time of physical activity [50]. With regard to smoking behaviors, participants were asked about the number of waterpipes smoked per week, the number of cigarettes smoked per day, and the number of smokers in the house.

The second part included the following scales:

*Lebanese Waterpipe Dependence Scale (LWDS-11)*. This previously validated instrument in Lebanon was used to evaluate the waterpipe smoking dependence status [51]. The total score was computed by summing the answers of all questions. Scores < 10 would indicate low dependence, whereas scores of 10 or more would indicate high dependence [52]. The McDonald’s omega value for the Lebanese Waterpipe Dependence Smoking-11 (LWDS-11) scale was 0.80.

*Depression, Anxiety and Stress Scale (DASS-8).* The 8-item screening tool was used to assess psychological distress. Items are scored on a four-point Likert scale, with higher scores indicating more psychological distress [53]. In the present study, the McDonald’s omega value was 0.88.

*Waterpipe Harm Perception Scale (WHPS).* This scale is composed of 6 items comparing waterpipe harm to that of cigarettes [54]. The response options for these six questions were as follows: less (1); equal (2); and more (3). A higher score value indicates that WP is perceived as more harmful. In the present study, the McDonald’s omega value for the WHPS was 0.86.

*Waterpipe Knowledge Scale*. This scale is composed of nine questions validated in Lebanon and assessed based on a 5-point Likert scale, ranging from 1 to 5 (1 = strongly disagree, 2 = disagree, 3 = neutral, 4 = agree, 5 = strongly agree) [55]. Examples of the asked questions are “waterpipe is more harmful than cigarette”, “waterpipe contains components that may cause cancer”, “waterpipe is safe because it is filtered”, “waterpipe contains addictive substances”, and “waterpipe affects the fetus in pregnant women”. A total knowledge score was created by summing up the nine questions. The score ranged from 9 to 45. Higher score would indicate higher knowledge about the harmful effect of WPS. The McDonald’s omega value was 0.87.

*Waterpipe Attitude Scale.* This scale is composed of eight questions on participants’ attitude and validated in Lebanese population [55]. These questions were measured on a 5-point Likert scale, ranging from 1 to 5 (1 = strongly disagree, 2 = disagree, 3 = neutral, 4 = agree, 5 = strongly agree). Examples of the asked questions are “Support smoking ban for those under the age of 18”, “Support smoking bans in restaurants”, “Support smoking bans on transportation”, and “Support smoking bans in public parks”. A total attitude score was created by summing up the eight questions. The score ranged from 8 to 40. A higher score would indicate a more positive attitude toward WPS ban. The McDonald’s omega value for the attitude scale was 0.94.

*Fagerstrom Test for Nicotine Dependence (FTND).* The test is based on six elements that assesses cigarette dependence [56]. The higher the FTND score, the more intense the physical nicotine dependence. The scale has been previously validated in Lebanon [57]. In the present study, the McDonald’s omega value for the FTND was 0.68.

*Motivation to Stop Scale*: Forward and back translation was performed to translate this scale from English to Arabic. A certified translator translated the English version to Arabic, which in turn was re-translated back to English by a different translator. Upon completion of this process, the translators paralleled the English versions of the scale to assess whether the variables had the same meaning. It is a one item with seven response categories ranging from 1 (lowest) to 7 (highest level of motivation to stop smoking) [58]. This instrument incorporates intention, desire and belief to quit smoking and the following questions were asked: “Which of the following best describes you?” Response options were: “I don’t want to stop smoking” (1), “I think I should stop smoking but don’t really want to” (2), “I want to stop smoking but haven’t thought about when” (3), “I really want to stop smoking but don’t know when I will” (4), “I want to stop smoking and hope to soon” (5), “I really want to stop smoking and intend to in the next 3 months” (6), and “I really want to stop smoking and intend to in the next month” (7). At the end, participants were categorized into two categories: (1) No motivation to quit and (2) Motivated to quit.

The third part of the survey included questions about whether the warnings are efficacious to stop smoking (in your opinion), if the participant has been affected by warnings on tumbac boxes, the importance of health warnings on tumbac boxes in his/her opinion, if the warnings increased curiosity for information about how to stop waterpipe smoking, if the pictorial warnings are more effective than textual ones in your opinion, if the participant would consider changing tumbac brand if company uses pictorial warnings, and about his preferred choice of warnings (textual, pictorial or both).

### Statistical analysis

Data entry and analysis were performed on Statistical Package for the Social Sciences (SPSS) software, version 23. McDonald’s omega was calculated for the different scales used in this study to check their reliability. Binary logistic regressions were performed between each independent variable and the dependent variable (motivation to quit vs. not) (unadjusted models). Afterwards, the same analyses were repeated after adjusting over all confounders (adjusted models). Statistical significance was set at *p* < 0.05.

## Results

### Sociodemographic and other characteristics of the participants

A total of 294 adolescents completed the survey, with a mean age of 17.71 ± 0.81 years and 64.6% females. The mean LWDS score was 12.49 ± 6.71, with 61.2% of the sample being waterpipe dependent (scores ≥ 10). In addition, 43.5% of those adolescents were not motivated to quit waterpipe smoking, whereas 45.9% were motivated to quit but did not set a time frame for it. Other characteristics of the participants are summarized in Table 1.

**Table 1:** Sociodemographic and other characteristics of the participants

	Total (n = 294)	Iraq (n = 123)	Lebanon (n = 171)	*p*
**Sex**				**< 0.001**
Male	190 (64.6%)	34 (17.9%)	156 (82.1%)	
Female	104 (35.4%)	89 (85.6%)	15 (14.4%)	
**Smokers inside the house**				**< 0.001**
No	94 (32.0%)	22 (23.4%)	72 (76.6%)	
Yes	200 (68.0%)	101 (50.5%)	99 (49.5%)	
**Cigarette smoking**				**0.001**
No	194 (66.0%)	94 (48.5%)	100 (51.5%)	
Yes	100 (34.0%)	29 (29.0%)	71 (71.0%)	
**Warning efficacious to stop smoking**				0.456
Not at all	164 (55.8%)	66 (40.2%)	98 (59.8%)	
A little	86 (29.3%)	34 (39.5%)	52 (60.5%)	
Moderately	33 (11.2%)	18 (54.5%)	15 (45.5%)	
Very	11 (3.7%)	5 (45.5%)	6 (54.5%)	
**Been affected by warnings on tumbac boxes**				0.455
No	223 (75.9%)	96 (43.0%)	127 (57.0%)	
Yes	71 (24.1%)	27 (38.0%)	44 (62.0%)	
**Importance of health warnings on tumbac boxes**				0.331
Not at all	27 (9.2%)	15 (55.6%)	12 (44.4%)	
A little	76 (25.9%)	28 (36.8%)	48 (63.2%)	
Moderately	99 (33.7%)	44 (44.4%)	55 (55.6%)	
Very	92 (31.3%)	36 (39.1%)	56 (60.9%)	
**Warnings increased curiosity for information about how to stop waterpipe smoking**				0.073
Not at all	95 (32.3%)	43 (45.3%)	52 (54.7%)	
A little	98 (33.3%)	47 (48.0%)	51 (52.0%)	
Moderately	55 (18.7%)	21 (38.2%)	34 (61.8%)	
Very	46 (15.6%)	12 (26.1%)	34 (73.9%)	
**Pictorial warnings are more effective than textual ones in your opinion**				0.256
No	76 (25.9%)	36 (47.4%)	40 (52.6%)	
Yes	218 (74.1%)	87 (39.9%)	131 (60.1%)	
**Consider changing tumbac brand if company uses pictorial warnings**				0.349
No	108 (36.7%)	49 (45.4%)	59 (54.6%)	
Yes	186 (63.3%)	74 (39.8%)	112 (60.2%)	
**Choice of warnings**				0.214
Textual only	50 (17.0%)	19 (38.0%)	31 (62.0%)	
Pictorial only	26 (8.8%)	15 (57.7%)	11 (42.3%)	
Textual and pictorial	218 (74.1%)	89 (40.8%)	129 (59.2%)	
**Motivation to Stop Scale categories**				**0.024**
No motivation to quit	128 (43.5%)	63 (49.2%)	65 (50.8%)	
Motivated to quit	166 (56.5%)	60 (36.1%)	106 (63.9%)	
Age (years)	17.71 ± 0.81	17.41 ± 0.97	17.92 ± 0.58	**< 0.001**
Household crowding index (person/room)	1.38 ± 0.81	1.27 ± 0.62	1.45 ± 0.92	**0.045**
Physical activity index	27.05 ± 20.91	25.02 ± 21.43	28.51 ± 20.59	0.160
Number of smokers in the house	1.92 ± 1.36	2.36 ± 1.61	1.60 ± 1.04	**< 0.001**
Psychological distress	8.98 ± 6.10	9.37 ± 6.47	8.70 ± 5.82	0.358
Waterpipe harm perception	12.37 ± 3.23	12.15 ± 3.26	12.54 ± 3.21	0.306
Knowledge about waterpipe	29.66 ± 5.93	29.00 ± 6.25	30.13 ± 5.67	0.106
Attitude towards waterpipe	29.24 ± 7.18	28.41 ± 7.51	29.85 ± 6.88	0.089
Cigarette dependence	1.58 ± 2.80	1.12 ± 2.48	1.91 ± 2.97	**0.014**
Waterpipe dependence	12.49 ± 6.71	12.51 ± 6.75	12.47 ± 6.69	0.956

Numbers in bold indicate significant *p* values. Numbers are displayed as frequency (percentage) or mean ± standard deviation

### Bivariate and multivariable analysis

The results of the bivariate analysis results (unadjusted model) showed that compared to not at all, participants who think warnings are a little (OR = 2.40), moderately (OR = 2.66) and very (OR = 5.21) efficacious to stop smoking had motivation to quit. Having been affected by warnings on tumbac boxes compared to not was associated with motivation to quit. Compared to not at all, participants who acknowledged that warnings increase curiosity for information about how to stop waterpipe smoking a little (OR = 3.23), moderately (OR = 3.14) and very (OR = 5.08) had more motivation to quit. Finding pictorial warnings more effective than textual ones to stop smoking (OR = 2.04) and considering changing tumbac brand if company uses pictorial warnings (OR = 2.77) were associated with more motivation to quit. Finally, compared to textual warnings alone, choosing both textual and pictorial warnings on tumbac boxes (OR = 1.95) was significantly associated with motivation to quit.

When adjusting the results over confounding variables, the results showed that compared to finding the warnings to stop smoking not efficacious at all, adolescents who find the warnings moderately (aOR = 2.83) and very (aOR = 6.64) efficacious had higher motivation to quit. Compared to finding the warnings not increasing their curiosity for information about how to stop waterpipe smoking at all, participants who confessed that warnings increased their curiosity a little (aOR = 2.59), moderately (aOR = 3.34) and very (aOR = 3.58) had higher motivation to quit. Compared to not considering changing the tumbac brand if the company uses pictorial warnings, adolescents who would consider changing the tumbac brand (aOR = 2.15) had higher motivation to quit (Table 2).

**Table 2:** Bivariate and multivariable analyses of factors associated with motivation to quit vs. not*

	Unadjusted model (OR)	*p*	Adjusted model (aOR)	*p*
*Warning efficacious to stop smoking*		**0.001**		**0.030**
Not at all	1		1	
A little	2.40	**0.002**	1.91	0.055
Moderately	2.66	**0.017**	2.83	**0.045**
Very	5.21	**0.038**	6.64	**0.037**
*Been affected by warnings on tumbac boxes*				
No	1		1	
Yes	2.62	**0.001**	1.30	0.508
*Importance of health warnings on tumbac boxes*		0.186		**0.018**
Not at all	1		1	
A little	1.65	0.266	1.14	0.805
Moderately	1.06	0.901	0.34	0.053
Very	184	0.168	0.60	0.348
*Warnings increased curiosity for information about how to stop waterpipe smoking*		**< 0.001**		**0.013**
Not at all	1		1	
A little	3.23	**< 0.001**	2.59	**0.009**
Moderately	3.14	**0.001**	3.34	**0.008**
Very	5.08	**< 0.001**	3.58	**0.011**
*Pictorial warnings are more effective than textual ones in your opinion*				
No	1		1	
Yes	2.04	**0.008**	0.96	0.920
*Consider changing tumbac brand if company uses pictorial warnings*				
No	1		1	
Yes	2.77	**< 0.001**	2.15	**0.029**
*Choice of warnings*		0.058		0.786
Textual only	1		1	
Pictorial only	1.09	0.858	0.76	0.507
Textual and pictorial	1.95	**0.035**	1.04	0.937

*Reference group; OR = unadjusted odds ratio; aOR = adjusted odds ratio. The adjusted model is considered in terms of the following confounders: country, age, gender, smoking inside the house, psychological distress, waterpipe harm perception, knowledge about waterpipe, attitude towards waterpipe, cigarette dependence and waterpipe dependence and the other variables related to pictorial warnings. Numbers in bold indicate significant *p* values

## Discussion

Our study aimed to assess the association of textual versus pictorial warnings on tumbac boxes and the motivation to quit waterpipe smoking among adolescents located in two Eastern Mediterranean countries Lebanon and Iraq. Our results showed that adolescents who find that the warnings are efficacious to stop smoking had higher motivation to quit. Furthermore, participants who confessed that warnings increased their curiosity for information about how to stop waterpipe smoking had higher motivation to quit. Also, our results showed that adolescents who would consider changing the tumbac brand if the company uses pictorial warnings had higher motivation to quit.

### Association of health warning labels on motivation to quit smoking

This study demonstrated that adding the warnings on the tumbac boxes is associated with higher motivation to quit smoking both with and without a specific time frame, which is consistent with previous study [59]. The effectiveness of adding the HWLs on the waterpipe products to quit smoking has been shown in different studies to be an effective tool to quit smoking since it communicates the harms of waterpipe smoke among young age group [41, 60]. According to Süssenbach et al., HWLs on tobacco products serve as a crucial tool in the motivation to quit smoking since it increases awareness of the health risks of smoking such as lung cancer, heart disease, and other serious illnesses [61]. Our results highlighted that the addition of the labels increases warning visibility, reduces intent for waterpipe smoking initiation, and increases willingness to quit [2]. It has been further emphasized that the addition of HWLs has provided a strong evidence to be considered an effective tool to aid in smoking cessation since they are powerful techniques on conveying the risks associated with smoking [18]. Furthermore, the addition of HWLs on smoking products increases the motivation to quit, as these warning labels contribute to broader public health messaging campaigns and raise the awareness not only on an individual level but on a more broader basis mainly on the society level [62]. Therefore, the addition of HWLs that deliver and emphasize both threat and efficacy messages are considered effective communication tools and increase the motivation to quit smoking [63].

### Role of addition of pictorial warnings on the motivation to quit waterpipe smoking

Our results supported that implementation of pictorial warning was associated with higher motivation to quit waterpipe smoking among adolescents which is consistent with the results of other studies conducted in Lebanon and Jordan [30, 43]. Our results can be explained by the fact that our study involved young age participants who are concerned by the detrimental consequences of waterpipe tobacco smoking on reproductive disorders, development of non-communicable diseases, and cancer [60]. Furthermore, our study showed that waterpipe smokers tend to change the brand if the company uses pictorial warnings, which supports the fact that changing the content or rotating different warnings appears to renew attention and motivate towards quitting smoking [64]. It has been documented that pictorial warning exerts behavioral influence, disseminates the health information, contributes to raising the public awareness, and motivates smokers to quit smoking [65, 66].

## Clinical implications

Our study results highlight the need to implement HWLs, mainly pictorial, on all tobacco products, including waterpipe, in Lebanon. They also suggest a modification of the current labels in Iraq, in a way that pictorial warnings be placed on the water tube, promoting its visibility to smokers. Furthermore, HWLs should be added in an effective way that aim to increase exposure and deliver the message in an appropriate and effective intervention. It is of particular importance that labels should be placed on the waterpipe device or mouth piece as waterpipe adolescents who smoke in cafes are not exposed to HWLs [67].

## Limitations

Limitations include the possibility of response bias due to self-reporting, as well as selection bias (males outnumbered females) due to the use of convenience sampling and the fact that the sample included only smokers from cafés who may not be representative of the entire young population. Residual confounding bias is also possible, since there are other factors (such as age of waterpipe initiation and comparison between the different kinds of pictorial warnings on the motivation to quit) that were not measured in this study. In addition, our study did not assess other factors that might influence smoking tobacco products, such as the peer pressure and social belonging. Indeed, it was documented that peers influence each other to engage in waterpipe smoking sessions through the desire to conform with the feeling of belonging with the community and be part of the dynamic group [7]. We did not ask about the waterpipe smoking session length, which would have better reflected dependency.

## Conclusion

The present findings highlight that health warning labels and picture have positive influence on enhancing the need to quit waterpipe and tobacco smoking practice. Health warning themes should be placed on the waterpipe devices in an easily accessible and visible way to effectively deliver the message. This suggests the need for targeted messaging and interventions when addressing intention to quit waterpipe smoking among adolescents. More research is needed to elucidate the underlying factors associated with increased motivation to quit in response to HWLs.

## Data Availability

All data generated or analyzed during this study are not publicly available to maintain the privacy of the individuals’ identities. The dataset supporting the conclusions is available upon request to the corresponding author.
